# Developing a Work Accommodation Operating Model for Workplaces and Work Ability Support Services

**DOI:** 10.1007/s10926-024-10193-z

**Published:** 2024-04-16

**Authors:** Nina Nevala, Irmeli Pehkonen, Maarit Virtanen, Pauliina Mattila-Holappa, Pirjo Juvonen-Posti

**Affiliations:** 1 Finnish Institute of Occupational Health, Box 40, 00032 Työterveyslaitos, Helsinki, Finland; 2 Oulu University of Applied Sciences, Box 222, 90101 Oulu, Finland

**Keywords:** Disability, Implementation, Operating model, Rehabilitation, Work ability, Work accommodation

## Abstract

**Purpose:**

Workplace accommodation can help employees with disabilities remain employed or access employment rather than leave the labor force. However, the workplace accommodation process is still poorly understood and documented.

**Aim:**

The aim of this study was to develop a national operating model to make workplace accommodation interactive and procedural for workplaces and work ability support services and lower the threshold to making accommodations.

**Methods:**

The collaborative development process was carried out by a multiprofessional expert team with eleven workplaces in the private and public sectors in Finland. The design of this study was conceptual and developmental. The development process of the operating model consisted of four phases: the orientation phase, the joint planning phase, the implementation advancement phase, and the instilling phase.

**Results:**

The operating model has six stages: 1) identifying needs, 2) gathering knowledge, 3) exploring alternatives, 4) selecting solutions, 5) implementing solutions, and 6) monitoring and evaluating. The model defines the actions, roles, and responsibilities for each phase. To help implement the model, we published an information package, a guide, a planning formula, and a video animation in Finnish and Swedish.

**Conclusion:**

The operating model is a tool that workplaces and work ability support services can use to help working-aged people remain employed or access employment. Future studies should determine the workplace-specific functionality of this model using implementation research.

## Introduction

Previous studies have provided evidence that work accommodations are an effective way to promote and maintain employment despite reduced work ability among the working-aged [1–3]. Work accommodations can also increase innovation and productivity at the workplace [4, 5].

Work accommodations represent an important strategy for employers to support the work ability and employment of people with disabilities such as mental health problems [6–10], musculoskeletal disorders [11], physical disabilities [2, 12], developmental disabilities [13], chronic conditions [14–16], and aging [17]. Accommodation typically involves adjustments to work schedules, work processes and tasks, or work environments, which enable people to work despite disabilities, illnesses, living circumstances, or cultures [2, 3, 15, 16, 18]. The need for work accommodation can be either transient or sustained [13–16].

Despite the obvious benefits of these accommodations, they are not widely implemented [8], and needs for such accommodations often go unmet [19]. Employers may have several reasons for this, such as a lack of knowledge about different disabilities, accommodation solutions, and accommodation processes, or a fear of the costs involved [2, 3, 16]. Moreover, work ability support professionals may have insufficient knowledge and skills to help employers and employees implement accommodations [7, 20]. The interactive nature of the accommodation process is particularly important in the decision-making phase when the employer decides whether to grant the request for accommodation [21]. Even though disclosure is a prerequisite for being granted accommodations, an employee’s decision to disclose disability and need for work accommodation to an employer or supervisor is difficult, especially for people with mental disabilities [22, 23], due to stigma, discrimination, or fear that the disclosure will affect their job performance [8, 23, 24].

The work accommodation process is social, interactive, and non-linear, shaped by organizational and political factors and collaboration between stakeholders [21, 25]. In several cases, the process requires multisectoral collaboration between workplaces and health care, rehabilitation, or employment services [6, 13, 21]. Previous research has shown that employers’ resources and communication with employees and other stakeholders influence their accommodation efforts [3, 20, 26].

Disability management policies and practices have been positively associated with the likelihood that supervisors will accommodate the work of employees with back injuries [11]. According to McGuire et al. [11] and Williams-Whitt et al. [27], return-to-work processes require clear, well internalized disability management processes. However, the workplace accommodation process is still poorly understood and documented [21, 25], particularly in cases of mental health problems [9].

Earlier models exist, such as the International Classification of Functioning (ICF) [28] and the Job Accommodation Network (JAN) [29]. According to ICF, disability results from the interaction of person and environment [16, 28]. In this study, ICF was used as a theoretical framework. The operating model for work accommodation is an operationalization of strengthening participation in work [16, 28]. We wanted to develop a national model with roles, responsibilities and working tools suitable for work ability management at workplaces and service systems of work ability support. The JAN model [29], developed and used especially in the USA, served as an practical example in defining the phases of the work accommodation operating model.

The aim of this study was to develop an operating model to make workplace accommodation interactive and procedural for workplaces and work ability support services and lower the threshold to making accommodations. The operating model was intended to: 1) strengthen organizational processes by helping organizations plan their own work accommodation models for the workplace, and 2) provide guidance to workplace actors on how to accommodate work individually. In general, development projects are effective when they bring about change and achieve their objectives. Impact assessment is based on understanding the intervention’s impact chain. For example, the expected impact can be examined using the I-O–O-I (Input-Output–Outcome-Impact) chain [30], according to which the work accommodation operating model is a resource (input) that provides information on how the work accommodation process works in the workplace, on the different actors’ roles and tasks, and on why the work must be modified. The actors in the workplace adopt the model and modify (output), for example, the work of an employee who has been absent for a long time due to illness. As a result, the employee can return-to-work more quickly (Outcome 1). The introduction of the model also enables both employers and employees to understand that this is an equal, sustainable way to act in the workplace (Outcome 2). In the longer term, this will lead to a reduction in sickness absences (Input 1) and increased equality (Input 2) in the workplace [30].

## Methods

The design of this study was conceptual and developmental [31, 32]. The development process was carried out by a multiprofessional team of experts whose areas of expertise were work ability and disability, work ability management, rehabilitation, occupational therapy, ergonomics, psychology, and medicine. These experts collaborated with eleven workplaces from the private and public sectors, located in different parts of Finland.

The co-creation methods consisted of surveys, workshops and joint meetings with the multidisciplinary project group, employers, employees, and occupational health service (OHS) professionals. Finally, the same workplaces and the project steering group piloted the operating model. This workplace accommodation operational model was developed in the Finnish context as part of the ESF-funded “Work-related rehabilitation to lengthen work careers” project in 2020–2023.

During the *orientation phase,* we collected information from previous scientific literature and internet sources, i.e., theoretical frameworks and definitions, previous operating models, reasons for accommodations, accommodation solutions and phases, roles and responsibilities in the accommodation processes, barriers and facilitators, funding opportunities, and legislation related to granting work accommodations. During this phase, we also collected data on work ability support practices, i.e., work accommodation, from 11 workplaces. The employers were selected from a larger pool of the employer clients of two pension fund administrations, who had been asked to participate. The employers had no pre-existing relationships with the research units.

We sent an electronic questionnaire (Webropol) to 720 employees in the 11 workplaces, to which 297 (41%) responded between October 2020 and February 2021. The survey’s aim was to analyze the current work ability support available at these workplaces. The respondents worked in private and public sector jobs, such as public administration, education, social and health care, industry, news media, and services. Every fifth (19%) respondent was under 35 years of age, every second (48%) was 35–54, and every third (33%) was over 55.

During the *joint planning phase*, we defined the structure and developed the model prototype through a workshop and co-creation with employees from the workplaces. In this phase, we collected the opinions of the participants on the preliminary version of the operating model in a three-hour online workshop held in April 2021. The workshop was for employers, superiors, and occupational health care professionals. The participants could register for the workshop after they had received the information and invitation. The workshop’s aim was to produce new ideas interactively and for the participants to develop the preliminary version of the operating model together. Fifty people from 11 workplaces participated in the workshop, which consisted of an introduction to the theme, a presentation of the preliminary operating model, and teamwork to complete the preliminary model. The workshop participants were asked to discuss the issues in groups and take notes on whether each phase of the model included the key facts. Finally, the outputs were discussed, and the next steps of development were defined.

As *implementation advancements*, we asked the workplaces (employers or superiors as representative) and the project steering group to pilot the operation model and to fill out a short online questionnaire. First, we asked them to read the content of the operating model using an electronic platform (Howspace), and after this to write down the good points and those that should be corrected. Then, the operating model was corrected and published as open learning material in Finnish.

During the *instilling phase*, we created an implementation plan that contained the actions, roles, and responsibilities for instilling the operating model and the new tools at the workplace. Two public workplaces incorporated the operating model into their disability management process. We helped them implement the operating model by arranging meetings with the employers and superiors, and by commenting on their confidential strategic plans. With their permission, we published one case description of the implementation of the workplace accommodation operating model in the workplace.

## Results

### Orientation Phase

Several theoretical frameworks have been used to address work accommodation (Table 1). The frameworks of equality [33, 34], work ability and employment [35, 36], the International Classification of Functioning (ICF) [28, 37], rehabilitation [38], and ergonomics [39, 40], have all been reported in work accommodation studies. In Finland, legislation on equality, work safety, occupational health, and rehabilitation takes work accommodation into account.

**Table 1:** Frameworks used in work accommodation studies

Framework	Orientation of framework	Reference
Framework of equality	The framework of equality sees work as a human right, as well as reasonable accommodations, and the development of diversity in the workplace	[33, 34]
Framework of work ability and employment	This framework sees work accommodation as a part of the work ability concept, work ability management, work ability support, and employment. It also considers individual aspects other than health, such as competence, motivation and values, and environmental aspects such as work environment and management	[35, 36]
Framework of the International Classification of Functioning (ICF)	This framework identifies and classifies the domain of environmental factors, including work accommodation, as one of its health-related domains	[16, 28, 37]
Framework of work-related rehabilitation	The framework of work-related rehabilitation regards work accommodation as work ability support through multisectoral and multiprofessional cooperation at workplaces	[38]
Framework of ergonomics	The framework of ergonomics, especially macro-ergonomics and participative ergonomics, defines the workplace variables (i.e., accommodation) that can affect the work, work processes, and work ability support of employees	[39, 40]

During the orientation phase, we found a Job Accommodation Network (JAN) operating model [29, 41], on the basis of which we refined the six phases of our model: 1) Identifying needs, 2) Collecting knowledge, 3) Examining alternatives, 4) Choosing solutions, 5) Implementing solutions, and 6) Monitoring and evaluating.

Although stakeholders have different knowledge and responsibilities within the disability management process, their roles and expected actions remain poorly defined and understood [11, 42–44]. Therefore, effective management policies and practices are essential to reduce the duration and costs of disability [7, 11]. In workplaces, supervisors play a central role in work ability management processes such as work accommodations, because they are most familiar with the job requirements and have the mandate to implement accommodations [11]. They are usually the first people to communicate with employees and play a key role in the interface between the workplace and other stakeholders, such as employees, health care providers, and co-workers [43]. Workplace accommodation policies and practices should be clear, and supervisors’ perceptions of them should be strong; this makes the supervisors more likely to be supportive of accommodations [11].

The barriers to and facilitators of workplace accommodations are often related to job, employee, workplace, and accommodation factors (Table 2). For employers, the most notable barriers to providing accommodations are a lack of knowledge and capacity [2, 21, 33], inadequate assessment procedures [25], a lack of collaboration between different actors [3], and a fear of costs [2, 5]. For employees, the most notable barriers are a lack of knowledge about workplace accommodations and a fear of disclosing disabilities [2, 5].

**Table 2:** Scientific knowledge used to develop the operating model of work accommodation operating model

Theme of scientific knowledge on work accommodation	References
Culture, communication, and attitudes in the workplace	[24, 25, 48–50]
Utilization of work accommodation at different stages of employees’ working careers	[3, 36]
Disclosing and requesting accommodations, user needs	[2, 33, 51]
Work accommodation process with roles and responsibilities of various stakeholders	[11, 25, 33, 42–44, 48, 49]
Knowledge of work accommodation, sources of information	[27, 33]
Cooperation and liaison between different actors	[20, 37, 47]
Categorization of accommodation solutions	[2, 8, 16, 52]
Costs and benefits of work accommodations for employers	[5, 41]
Barriers to and facilitators of work accommodations	[2, 14, 20, 21, 33, 42]

According to the workplace survey (*n* = 297), less than half (45%) of the respondents either strongly or somewhat agreed with the statement “There is enough knowledge and skills to accommodate work in my workplace” (scale 1 = strongly agree, 5 = strongly disagree). Every sixth (15%) respondent had personal experience of work accommodation. Most of these workers (*n* = 45) who had received accommodations either strongly agreed or somewhat agreed with the statements that they were active participants in the accommodation process (80%), the accommodations were beneficial for their work ability (80%), the supervisor was actively involved in the process (73%), the accommodations were beneficial (67%), and the accommodation process was smooth (65%).

Every third (35%) of respondents considered the accommodation process smooth. The most common accommodation solutions were changes in work organization (63%), work schedules (43%), and the work environment (42%).

### Joint Planning Phase

In the workshop, we listed several areas of the preliminary operating model that required improvement (Table 3). The participants were workplace actors and members of the project’s steering group.

**Table 3:** Areas of the work accommodation operating model that required improvement determined in the workshop (*n* = 50)

Phase	Areas requiring improvement
Identifying needs	The operating model should be presented to the work groups and all employees and supervisors should be made familiar with it. This makes it easier to identify needs for accommodation
	Needs for accommodation can be identified by various stakeholders such as occupational physiotherapists, occupational psychologists, or other OHS professionals
	Collaboration with different stakeholders is essential
	The competence and motivation of employees, supervisors, and service providers should be carefully considered
Collecting knowledge	The supervisor must know the employee’s work tasks
	Information on work can be obtained by, e.g., surveys
	Employees’ wishes should be considered
	Each workplace should have a list of possible accommodation solutions
Examining alternatives	Alternative tasks should be considered. Joint work, such as planning, administrative tasks, undone work, and training, is usually neglected in everyday routines
	Work should be realistically designed
Choosing solutions	Collaboration among employees, supervisors, teams, and HR is important for selecting options
	Professional confidentiality is essential
	The diversity of workplace needs and opportunities (time- and place-bound work) should be considered
Implementing solutions	Roles should be clarified, especially concerning implementation
Monitoring and evaluating	The rest of the team should be listened to and considered during the assessment phase
	The assessment should be carried out systematically and in time
	The assessment should be documented

### Implementation Advancements

**Fig. 1:**
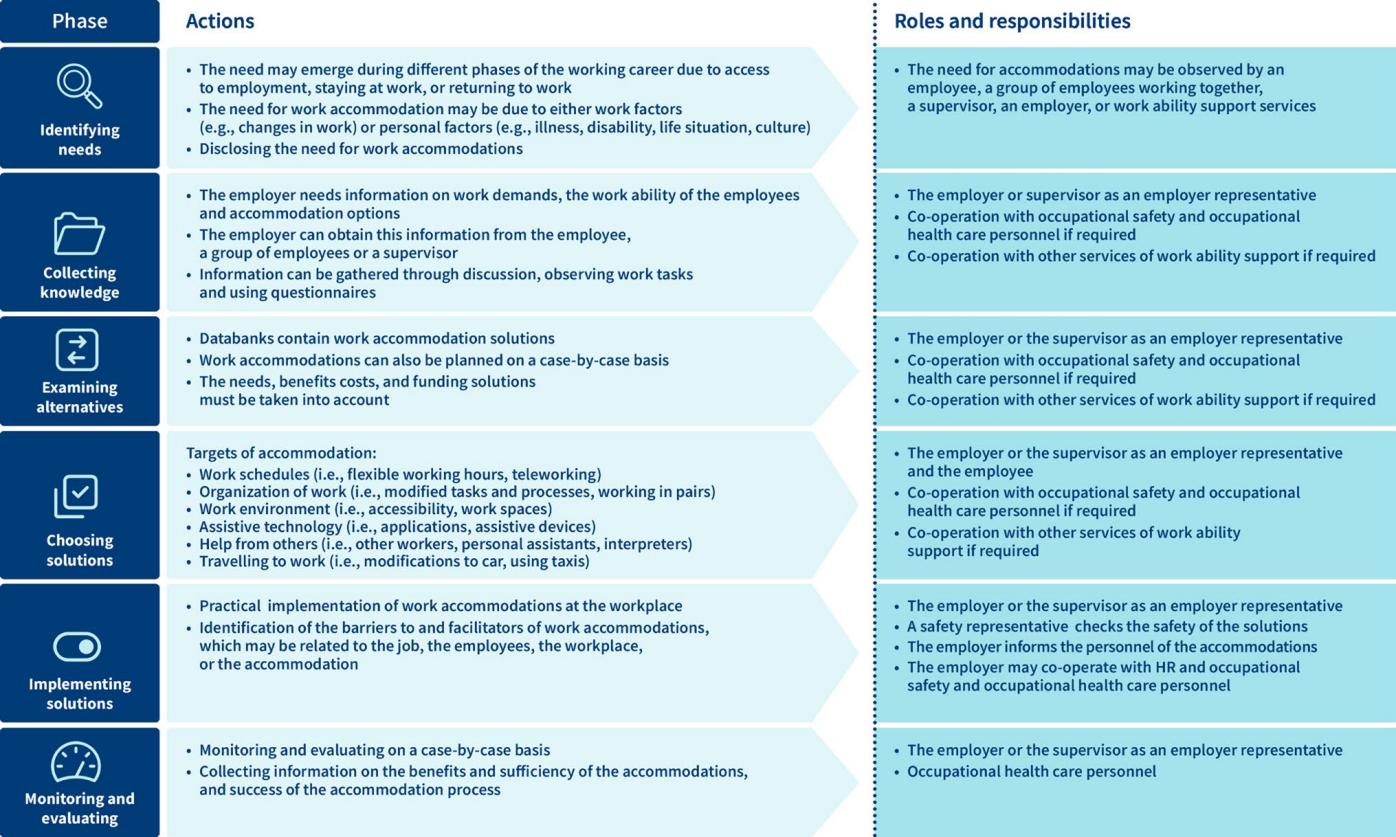
Work accommodation operating model’s six phases with actions, roles, and responsibilities

The work accommodation operating model was revised and supplemented based on the feedback from the multisectoral team (employers or superiors, the steering group). The employers’ feedback on the piloting helped us define the structure and content of the operating model. This meant more detailed text, better defined concepts, more condensed content, practical case examples, and a diagram of the process, roles and responsibilities. The employers also wished for new implementation tools, and that all the materials would also be available in Swedish. The final model defined the actions, roles, and responsibilities during each phase (Fig. 1).

### Instilling Phase

To support the implementation of the operating model, four tools (an information package, a guidebook, a planning formula, and a video animation) were developed and published in Finnish and Swedish for the employers and work ability support professionals.

The *information package* consisted of open learning material on work-related rehabilitation. One of the elements of this information package was a work accommodation operating model. The *guidebook* (29 pages) covered all the phases of the work accommodation operating model, definitions, scientific knowledge on the effects of work accommodation, three cases, and the related legislation. It also included a list of questions that an employer and other stakeholders could use when planning their own work accommodation operating model for their workplace. The *planning formula* was a supervisor's tool for work ability management. It was intended for use when a supervisor and an employee plan work accommodations together. The planning formula could also be used in collaboration with the employer and work ability support professionals, i.e. occupational health care professionals. The formula followed the work accommodation operating model, and could be completed either electronically or on paper. The short *video animation* followed the work accommodation operating model.

These tools can be used in the induction of new employees, in work ability support during the different stages of the working career, in client counseling, in occupational rehabilitation, and in vocational training. Work ability support professionals can also use these tools in multisectoral and multiprofessional collaboration. During this phase, the operating model and the new tools were disseminated through social media, seminars, lectures, articles, blogs, and conference presentations, in accordance with the implementation plan.

### Case: Implementation of Work Accommodation Operating Model in Early Childhood Education Services

A work accommodation model was required in the early childhood education services of a Finnish city administration. Its creation was motivated by a desire to promote employees' ability to cope with their work or return-to-work after an illness, family leave, or some other reason.

The early childhood education services planned their own work accommodation operating model for their workplace. At the beginning of the project, a developmental team was formed in this city administration. The team consisted of various personnel groups, such as management, middle management, supervisors, and occupational safety and health representatives. The team put together a list of possible reasons for work accommodations. They also examined which measures were already in place at the workplace and how the work could be accommodated. They came up with the following: 1) rearranging working hours, such as shorter working days or weeks, 2) limiting the number of duties and arranging new ones, 3) work trials in accommodated work, 4) work aids, 5) making the work environment accessible, and 6) assistance of other people.

Factors related to employees, work communities and work processes were considered for each of these items. Then, the responsibilities and roles of the individuals participating in the work accommodation process were agreed on. The supervisor was responsible for the accommodation process and handling the practical arrangements. If needed, the supervisors cooperated with the OHS, HR management, the occupational safety and health personnel, and the elected employee representatives. The employer and the employee signed a written agreement on the work accommodation, which specified whether the need for accommodation was temporary or permanent, as well as when and how the situation would be monitored and assessed. The accommodation solutions could be amended as necessary on the basis of the assessment.

In this city administration, the work accommodations were beneficial for the employees, work communities, and the workplace as a whole. They resulted in a decrease in the number of sick leaves, less stigma, improved wellbeing, and a better atmosphere in the work community. The workplace confirmed that the planned measures were carried out in practice. Furthermore, the positive attitude toward work accommodation improved the image of the employer, which subsequently promoted the recruitment of new employees.

## Discussion

This project developed a national work accommodation operating model for workplaces and work ability support services. We gathered information on work accommodations, the processes involved, and the barriers to and facilitators of their implementation from scientific and other sources. We learned lessons from the JAN model, following the recommendations of Johnston and Helms [29]. For the Finnish context, we considered the roles and responsibilities of the national actors of the service system, the sources of knowledge, the means and benefits of work ability support, and the national legislation.

Work accommodation is known to support work ability and help employees remain in, return to, or access employment rather than leave the labor force. The operating model can be used in workplaces and work ability support services such as OHS, rehabilitation services, employment services, and vocational training to support the work ability and employment of people of working age. In this project, we addressed work accommodations through three theoretical frameworks: equality, seeing work as a human right, work ability and work ability management, and OHS and rehabilitation. Depending on the needs of the work-aged person, the work accommodation process can be implemented by either only the workplace, or the process may require multiprofessional and multidisciplinary cooperation among several service providers.

Previous literature has identified several barriers to implementing workplace accommodations. Typical barriers are a lack of knowledge about workplace accommodations, communication deficits, and a fear of costs [2, 3]. Knowledge, skills, and tools are needed in both the workplace and the multiprofessional networks of professionals who support the work ability and employment of people with disabilities. Many organizations also lack effective accommodation processes [4], making it difficult for employers, employees, and service providers to understand the process, roles and responsibilities related to accommodation. We have described the operating model in a process-like manner, providing information on what should be done, how it should be done, and who is responsible at each stage of the process. The operating model contains references that offer more information if required.

Moving from knowledge to action requires concrete, easy-to-use tools in workplaces and in work ability support services, as suggested by Ilmarinen [45]. This means clear accommodation policies, the use of operating models, awareness of the accommodation options in the workplace, and training to implement the accommodations. To bridge the “knowing to doing” gap as described by Gould-Werth et al. [3] and Rudstam et al. [46], we developed a model that includes a guide, a planning formula, and a video animation, to help the actors implement the model in workplaces and in work ability support services.

The strengths of this project were its theoretical framework, multiscientific research group, co-creation with the workplace actors, and multiple co-creation methods such as surveys, workshops, joint meetings, and piloting. Further, the workplaces were actively involved in the development process. First, the workplaces participated in the workshop and provided valuable practical information for development. Second, they gave comments on the first published version of the model. We made improvements to the operating model on the basis of the feedback from the workplace actors.

During the development process, two organizations adopted this operating model for use as part of their work management process. One limitation of the study is that we had no opportunity to gather user experiences of this model over the long-term. In addition, information on the needs of small businesses remained limited. In the future, this operating model should also be modified for small workplaces.

The work accommodation process can be compared to an organizational change process that must be actively managed and accompanied, as reported by Kensbock et al. [42]. However, supervisors often find it difficult to assign employees appropriate tasks, because they focus on work and lack medical information. Clinicians in turn focus on symptom reduction, and often lack sufficient data on job demands and workplace factors, as shown by Cao et al. [47]. This operating model can also be a tool for the employee to propose and negotiate the accommodations that they need.

This operating model can be used as a common tool in multisectoral and multiprofessional networks to support the work ability and employment of working-aged people. In order to meet the diverse needs of clients, professionals must work together across the administrative boundaries of organizations. However, this way of working requires multiprofessional competence such as mutual understanding, a common language, and common tools. To extend the working careers of employees, workplaces are important actors who can enhance the competence of the stakeholders in work-related rehabilitation and cooperation-based operating models. Hopefully, this operating model will be taken into use in workplaces, social and health services, rehabilitation, and employment services. It can also be integrated into study programs of the sector’s colleges and universities. As described in Table 2, socio-cultural issues such as culture, communication, and attitudes in the workplace have been found out to be both facilitators of and barriers to the implementation process [24, 25, 48–50]. We were unable to observe these phenomena in different workplace contexts in our remote co-development work. We recommend that implementation research be used in the future to identify workplace-specific functionality, socio-cultural factors included.

In the future, it would be important to use implementation research to identify workplace-specific functionality. This model can be used to increase knowledge, reduce the barriers to work accommodation and to strengthen the facilitators. It will hopefully increase multiprofessional and multisectoral cooperation and lower the threshold to work accommodations by making the accommodation interactive and procedural for workplaces and work ability support services. The model may also reduce stigma and make it easier for employees to disclose their conditions and needs, which is known to be a major barrier to initiating the accommodation process.

## Conclusions

The work accommodation operating model is a tool for workplaces and work ability support services to help working-aged people remain employed and access employment. Hopefully, the operating model will make accommodation interactive and procedural for workplaces and lower its threshold. In the future it would be important to determine the workplace-specific functionality of this model using implementation research.
